# Climate change, vaccines, GMO: The N400 effect as a marker of attitudes toward scientific issues

**DOI:** 10.1371/journal.pone.0273346

**Published:** 2022-10-06

**Authors:** Łukasz Okruszek, Aleksandra Piejka, Natalia Banasik-Jemielniak, Dariusz Jemielniak

**Affiliations:** 1 Social Neuroscience Lab, Institute of Psychology, Polish Academy of Sciences, Warsaw, Poland; 2 Institute of Psychology, The Maria Grzegorzewska University, Warsaw, Poland; 3 Management in Networked and Digital Societies (MINDS) Department, Kozminski University, Warsaw, Poland; University of Milano–Bicocca: Universita degli Studi di Milano-Bicocca, ITALY

## Abstract

While the psychological predictors of antiscience beliefs have been extensively studied, neural underpinnings of the antiscience beliefs have received relatively little interest. The aim of the current study is to investigate whether attitudes towards the scientific issues are reflected in the N400 potential. Thirty-one individuals were asked to judge whether six different issues presented as primes (vaccines, medicines, nuclear energy, solar energy, genetically-modified organisms (GMO), natural farming) are well-described by ten positive and ten negative target words. EEG was recorded during the task. Furthermore, participants were asked to rate their own expertise in each of the six topics. Both positive and negative target words related to GMO elicited larger N400, than targets associated with vaccines and natural farming. The results of the current study show that N400 may be an indicator of the ambiguous attitude toward scientific issues.

## Introduction

The recent decade has brought an unprecedented rise in anti-scientific and anti-intellectual movements in Western countries [1, 2]. The trust in science and the traditional hierarchies of knowledge is declining [2], and the process can be linked to the general growth of distrust in social institutions and governments [3]. According to some researchers, the increasing disregard for facts may be amplified by network propaganda [4]. According to others, the unprecedented detour from reason [5] may be rather linked to the organic development of the so-called collaborative society [6]: a phenomenon of people cooperating online to substitute the traditional knowledge sources and replacing formal expertise.

Collaborative society has democratized knowledge for the good and for the bad. The impressive growth of open knowledge movements, including Wikipedia [7] the explosion of popularity of self-proclaimed experts, visible e.g. in the reliance on “Doctor Google” in medicine [8], or the rapid development of alt-med support communities [9] have definitely contributed to the erosion of trust in professional expertise, as well as to the radicalization of some individuals and conspiracy theorists [10]. The recent European funded project “Communication role on perception and beliefs of EU Citizens about Science” (CONCISE), which aimed at investigating the beliefs, perceptions, and knowledge of four controversial scientific topics, i.e. climate change, vaccines, alternative medicine, and genetically modified organisms (GMO) technology, found that a plethora of different factors shapes public trust and distrust in different sources of science information [11]. In Poland, for example, the multitude of unreliable information that can be found in digital media, the perceived need to thoroughly verify the received information, and the vigilance towards many actors (such as politicians or journalists) make it extremely difficult to find a universal model for communicating science of all those topics in a way as to ensure its broad social reception [12].

Furthermore, investigation of factors underlying anti-scientific stance is particularly important in the era of COVID-19 crisis. Anti-scientific stance is widely perceived as one of the factors which may hinder COVID-19 vaccine effort, which is particularly dangerous in times of politicization of anti-scientific discourse [13]. Several vaccine-preventable infectious diseases have resurfaced due to the decreased vaccination-rates [14], and some countries are already falling below the threshold of herd immunity for preventable diseases, such as measles [15]. However, anti-scientific stand is not limited to medical issues. For instance, climate change denialism has significantly impacted public policies over the last decade [16, 17]. The controversies about climate-friendly energy result in massive protests and confusion [18]. Other pseudo-scientific theories have led to multiple 5G antennas being set on fire all over the world in 2020, as a result of conspiracy theories about their effect on health [19]. Given the significance of the antiscience stand for various societal outcomes, a lot of interest has been put recently into studying mechanisms that may underlie and sustain antiscience beliefs [20]. A large body of the social psychology literature has examined this issue, examining both explicit [21] and implicit [22] processes associated with anti- and pseudo-scientific beliefs.

The anti-science movement has been growing in recent years, as more and more people become skeptical of the claims made by experts in various fields. The movement has been particularly active in the areas of vaccination, nuclear energy, and genetically modified organisms (GMO) technology [23, 24]. In each of these areas, the anti-science movement has raised serious questions about the safety and efficacy of the technology or practices in question. While the anti-science movement has sometimes been dismissed as a fringe movement, its influence appears to be growing, as evidenced by the growing number of people who are skeptical of the claims made by experts in these fields.

It has been emphasized that social neuroscience methods allow for unobtrusive investigation of neural processes associated with belief formation, prejudice, and stereotyping [25, 26]. While many studies have used functional neuroimaging methods to investigate the architecture of brain networks associated with implicit belief formation [27], the advantages of using electrophysiology (EEG) and, particularly, event-related potentials (ERPs), to examine neural bases of belief formation have also been emphasized [28]. ERPs provide excellent temporal resolution, which allows for investigation of the time-course of processes involved in belief formation. Furthermore, EEG investigation can be performed outside the scope of the laboratory, thus it can be extended to more ecologically valid field-investigations. These factors stress out the importance of investigating the feasibility of use of EEG protocols to study belief formation.

One of the promising candidates for use as a marker of processes associated with belief formation is N400 evoked-potential. First described by Kutas and Hillyard [29], it was initially found in paradigms based on semantic incongruity effect and used mostly for linguistic research. However, soon it has been extended into the social neuroscience field and effectively applied for research on stereotyping [30] and prejudice [31]. Furthermore, increased N400 was shown to be elicited by the presentation of value-inconsistent words presented as a part of morality-oriented statements [32]. Previous research has also shown that N400-based methodology may be effectively applied, to study attitudes toward political issues [33]. Thus, the aim of the current study is to investigate whether N400 potential may be used for investigating the attitudes toward scientific issues, particularly vaccination, medicines, nuclear energy, solar energy, genetically-modified organisms and natural farming. Polish public opinion surveys have shown that nuclear energy is less acceptable compared to renewable sources of energy [89% of acceptance vs. 53% of acceptance: 34]. Similarly, over 65% of Polish respondents agree with an opinion that farming of GMO should be banned [35]. Furthermore, a rising tide of anti vaccination movements has been linked to the decreasing percentage of adults who perceive vaccinations as safe for children and this effect was observed long prior to the COVID-19 pandemics [36]. Taking these factors into consideration, we expect that science-related, polarizing topics will be appraised negatively on the behavioral level. Furthermore, we expect to find larger N400 amplitude to positive and smaller N400 amplitude to negative words describing factors which will be appraised in a more negative manner than other issues.

## Methods

### Participants

An opportunity sample of 31 young adults (22.65+/-1.25 y.o., 16F) was recruited for the participation in the current study via online social platforms and word-of-mouth invitations. Participants declared no history of psychiatric and neurological disorders. Only right-handed participants were included in the study. Prior to any procedures, participants provided informed written consent. The procedure of the study was approved by the Ethics Committee at the Kozminski University in Warsaw. Participants were reimbursed with a voucher that could be exchanged for cinema tickets (worth approximately 10 USD) upon completion of the EEG session. To avoid biasing our participants, we did not screen their scientific literacy, or pseudo-scientific beliefs prior to the main procedure. However, we asked our participants to provide additional information via an online Qualtrics-based questionnaire. Unfortunately, this led to some participants (7/31) not completing the additional assessment after leaving the laboratory.

### Paradigm

The paradigm for the current study was created on the basis of the previous studies that examined attitudes by using N400 [30, 37].

For the task, we picked three categories (medicine, food, energy sources) of which we chose three controversial science-related issues, analogous to those examined in the CONCISE project (vaccination, GMO technology, nuclear energy). We matched each of the issues with a topic from the same category. For that, we chose topics that are either appraised almost unanimously positively (solar energy) or do not provoke public debate (natural farming, solar energy).

During the task, participants were informed that he/she would be presented with a pair of words (vaccination (‘szczepienie ochronne’) / medications (‘przyjmowanie leków’) / GMO technology (‘technologia GMO’) / natural farming (‘rolnictwo naturalne’)/ nuclear energy (‘energia jądrowa’)/ solar energy (‘energia słoneczna’)) which would be followed by the adjective. Six types of primes (matched in the number of pronounced syllables) were presented, with each two corresponding to a certain category (medicines, food production, energy sources).

**Fig 1:**
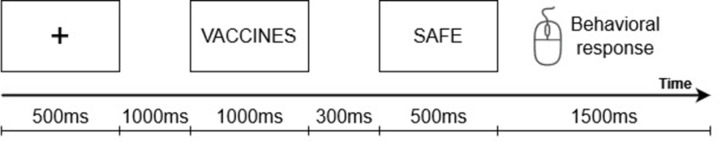
Schematic illustration of a trial in the EEG paradigm.

Each prime was presented in a written form for one second, and then after 300 msec a target word was presented for 500 ms. Ten positively valenced adjectives (effective / reliable / sure / useful / proven / efficient / needed / predictable / safe / healthy) and ten negatively valenced adjectives (ineffective / uncertain / useless / risky / dangerous / harmful / questionable / unnecessary / inefficient / unreliable) were presented as target words for each prime. Participants were asked to answer whether the target word accurately describes prime during 1500 ms after target word presentation. After one-second ITI the next trial started. The order of the prime/target words was pseudo-randomized to avoid presenting the same prime more than three times in a row. To produce enough trials, two runs of the task were presented, so each type by valence combination could have 20 trials. A schematic illustration of the procedure is presented in the Fig 1.

To ensure that the proposed paradigm can reliably elicit N400 and to compare the effects observed in the main task with semantic N400 effects, we also added a third run, where 30 nouns were paired with semantically congruent (‘scissors’–‘sharp’) or incongruent (‘scissors’–‘boiling’) adjectives. The list of adjectives was based on the Kent-Rosanoff Word Association Test, adopted for the Polish language [38] and can be found in S1 Table. The semantic N400 task was presented in the same manner and with the same timings as the main tasks upon completion of the two runs of the main task.

### EEG recording and analysis

EEG was recorded in the Institute of Psychology, Polish Academy of Sciences using Neuroscan SynampsRT amplifier and 64-channel QuikCap. Online signal was sampled at DC to 1000 Hz and recorded using Curry8 software. With very few exceptions, impedances were kept below 5 kOhms. Standard offline preprocessing was done using EEGlab [39] and ERPlab [40]) scripts. The signal was downsampled to 250 Hz, filtered with bandwidth 0.1–30 Hz and re-referenced to linked mastoids. Fragments of signals containing large non-stationary artifacts were manually removed prior to the ICA decomposition. Eye-blink components were then removed prior to epoching the signal into 1000 ms adjective-locked epochs, which included 800 ms of signal post-stimulus onset and 200 ms baseline, which has been used for baseline correction. Any residual artifacts which would result in abnormally large signal (over +/-100 μV) were rejected prior to the ERP extraction. The Late Positive Component (LPC) in response to prime-words was extracted between 350 and 600 ms. post-stimulus onset from Pz electrode. The N400 mean value was extracted in response to target-words from four midline electrodes (Fz, FCz, Cz, CPz) between 350 and 450 ms post-stimulus onset. Data from all the participants who completed the EEG session were included in the analysis.

### Questionnaire assessment

After the EEG measurement, participants were asked to complete a short survey, which contained a question on whether they were previously acquainted with each subject presented during the EEG task (“yes/no”) and how they assess their own knowledge on each of the subjects (a seven-point Likert scale). Two participants did not provide this information.

Furthermore, during the post-hoc online assessment, subjects were asked to complete a task ([more details: 21])). Briefly, participants were presented with pseudo-scientific claims related to four different domains (climate change, GMO food, alternative medicine, psychological myths). For each domain, three pre-existing pseudo-scientific statements were collected from the webpages (e.g. “Global warming in recent decades has been associated with changes in solar activity”) and two additional myths were created for the purpose of the questionnaire (e.g. “Due to the radiation they emit, nuclear power plants are as much a contributor to climate change as coal power plants”). For each of the statements, participants were asked to decide whether they have previously encountered such a claim. Furthermore, a five-point Likert scale was provided [Definitely no (1)–Definitely yes (5)] and participants were asked to answer to what level they found the statement to be true. Additionally, to examine participants’ scientific literacy, participants completed the 9-item Scientific Literacy Scale (SLS), adapted from [41].

### Statistical analysis

Repeated-measures ANOVA with within-subject factors Type (six types of primes) and valence (two levels: positively valenced targets vs negatively valenced targets) were used to investigate both behavioral results of the task. As subjects predominantly perceived positive target words as congruent with primes (prevalent majority of ‘yes’ answers) and negative target words as incongruent (prevalent majority of ‘no’ answers) with primes, the main outcome measure for each prime was the percentage of ‘yes’ responses for positive targets and the percentage of ‘no’ responses for negative targets. To avoid scores being biased by trials without response, task-response scores were calculated for the number of congruent/incongruent responses divided by all the given responses for a given category. The LPC was analyzed using repeated-measures ANOVA with Type (controversial vs noncontroversial). The semantic N400 was analyzed with regard to the within-subject factor Type (two levels: congruent vs incongruent) and Electrode (four levels: Fz-FCz-Cz-CPz). N400 amplitudes from the main tasks were analyzed using repeated-measures ANOVA with within-subject factors Type (controversial vs noncontroversial), Valence (two levels: positively valenced targets vs negatively valenced targets) and Electrode (four levels: Fz-FCz-Cz-CPz). In both cases (LPC to primes, N400 to targets) follow-up analyses with six specific topics was also performed. Perceived expertise on each of the subjects was compared across categories using rm-ANOVA with six types of issues as a within subject factor. Post-hoc questionnaire responses were examined with rm-ANOVA to compare the mean level of perceived likelihood pooled over each five statements in four categories (climate, psychology, GMO, alternative medicine). All the post-hoc tests were Bonferroni corrected. Furthermore, to investigate the association between task-related behavioral results, subjective expertise on each issue and scientific literacy/pseudo-scientific beliefs we have used Pearson/Spearman correlations, which have been corrected to account for multiple comparisons. To avoid inflating the number of corrections we inspected only issue-specific correlations, e.g. expertise on specific issues was investigated with regard to specific behavioral/ERP scores (thus producing 2 comparisons and p = .025). For the general scores (SLS, pseudo-scientific beliefs) the correction for the full set of 12 comparisons was applied.

## Results

### Behavioral paradigm

**Fig 2:**
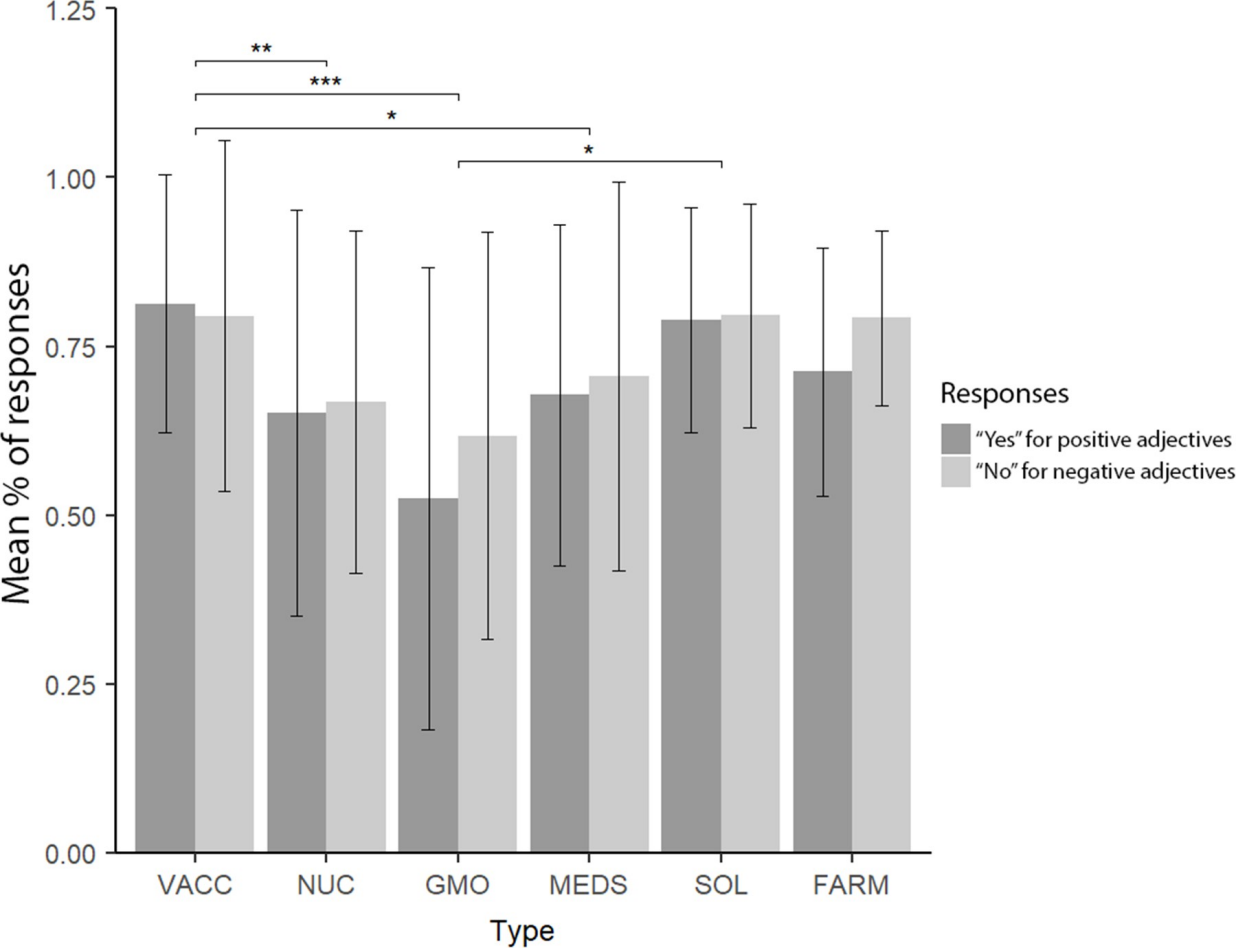
Behavioral responses to categories in the task. VACC = vaccination, NUC = nuclear energy, GMO = GMO technology, MEDS = medicines, SOL = solar energy, FARM = natural farming. The percentage is presented for positive adjectives rated as congruent (“yes”) with a cue and negative adjectives rated as non-congruent (“no”). *** p < .001, ** p < .01, * p < .05.

The main effect of the type of the issue was observed (F(5,150) = 7.84, p < .001 η^2^_s_ = .207) with GMO being rated less favorably than vaccines (p < .001) and solar energy (p = .011). Furthermore, nuclear energy (p = .001) and medicines (p = .022) were rated less favorably than vaccines. No effect of valence (F(1,30) = 1.74, p = .197 η^2^_s_ = .055) or interaction between type and valence (F(5,150) = 1.87, p = .134 η^2^_s_ = .059) were observed. The results can be found in Fig 2.

### Semantic N400 analysis

**Fig 3:**
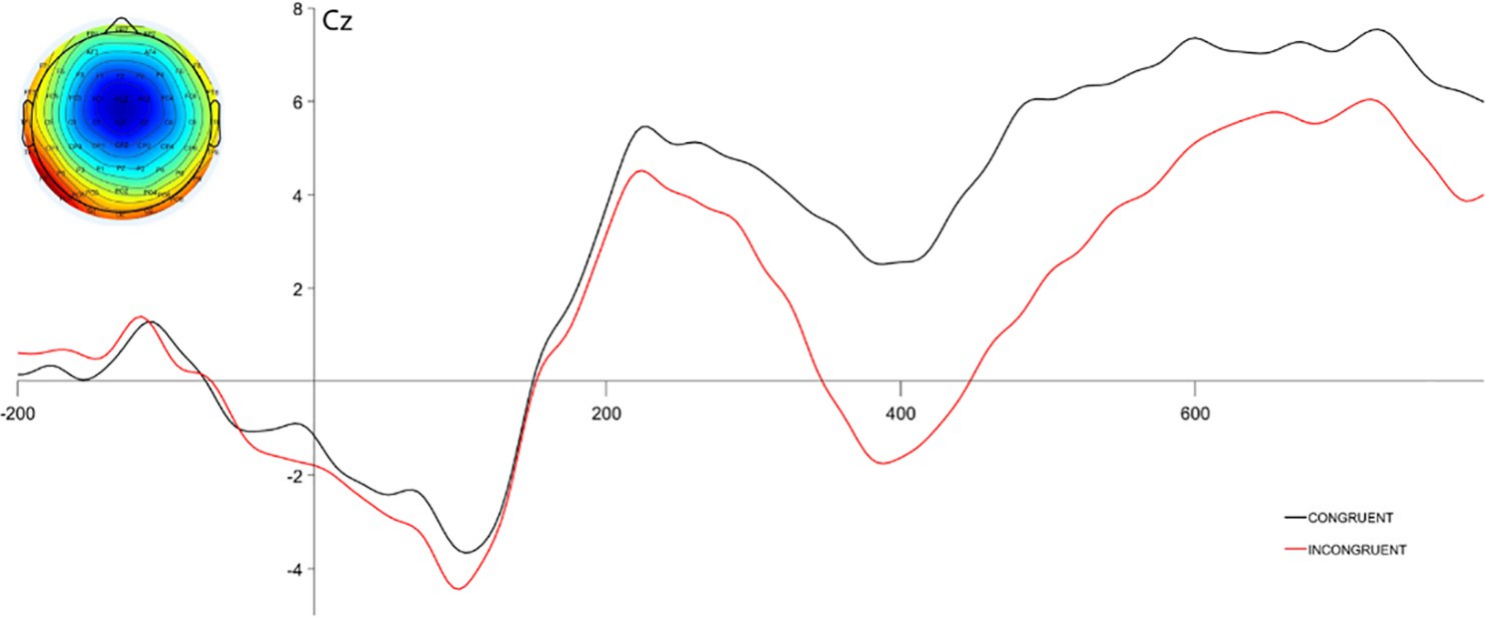
ERP waveform for semantic N400 paradigm observed on the CZ. Scalp map of the mean activity between 350 and 450 ms post-stimulus onset is presented in the upper left part of the figure.

In line with previous observations a robust effect of the type of the trial was observed (F(1,30) = 68.82 p < .001 η^2^_s_ = .696) with semantically incongruent adjectives eliciting significantly more negative N400 (-1.76+/-4.33 μV) than congruent adjectives (2.11+/-4.38 μV). Furthermore, the main effect of the electrode was observed, with (F(3,90)– 64.71, p < .001 η^2^_s_ = .683) with amplitudes of the signal rising from frontal to centroparietal sites. The results can be found in Fig 3.

### Attitude N400 analysis—ERPs to prime words

The pooled analysis revealed a significant main effect of the type of the prime words with controversial prime words (2.72+/-3.27 μV) eliciting larger LPC than noncontroversial prime words (1.96+/-3.14 μV; F(1, 30) = 11.34, p = .002 η2s = .274).

**Fig 4:**
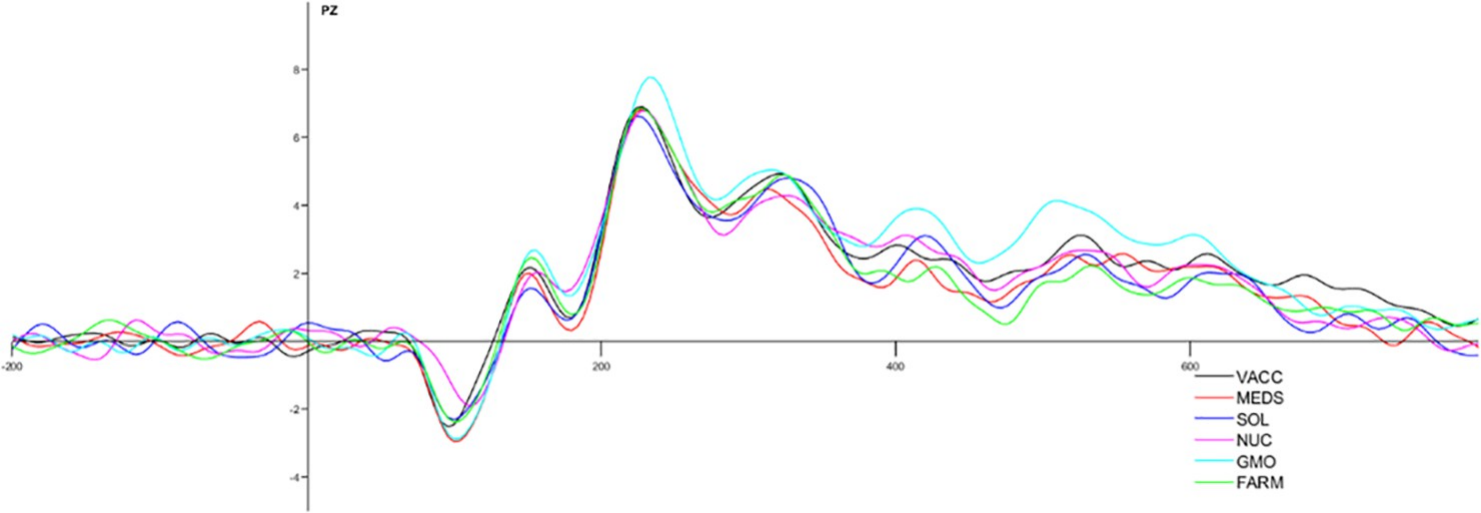
ERP waveform to prime-words for six conditions presented during the study on the PZ. VACC = vaccination, NUC = nuclear energy, GMO = GMO technology, MEDS = medicines, SOL = solar energy, FARM = natural farming.

In the analysis with separate topics, the main effect of the type of the issue was observed F(5, 150) = 3.85, p = .003 η2s = .114) with “GMO technology” eliciting larger LPC (3.25+/-3.36 μV) than pharmaceuticals (2.01+/-3.18 μV p = .018), solar energy (2.08+/-3.54 μV p = .032) and natural farming (1.77+/-3.37 μV p = .002). No other contrasts were significant. The results can be found in Fig 4.

### Attitude N400 analysis—ERP to target words

The pooled analysis revealed no significant task-related effects (type of the issue: F(1,30) = .53, p = .474 η^2^_s_ = .017; valence: F(1,30) = .36 p = .363 η^2^_s_ = .028) or interactions.

In the analysis with separate topics, main effect of the type of the issue was observed (F(5, 150) = 4.204, p = .001 η^2^_s_ = .123) with significantly more negative N400 amplitudes for adjectives following GMO-cue, than for vaccines (p = .010), and natural farming (p = .027). No other between-category comparisons were significant.

**Fig 5:**
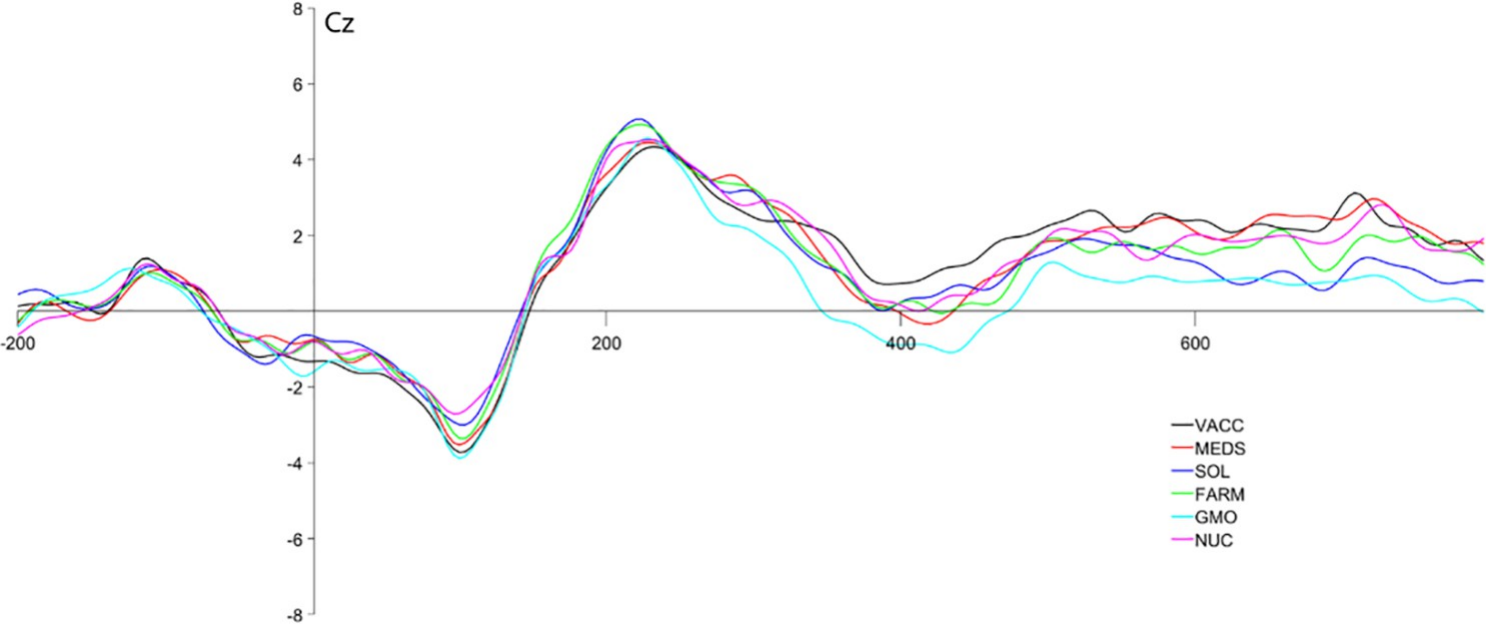
ERP waveform to target-words for six conditions presented during the study on the CZ. VACC = vaccination, NUC = nuclear energy, GMO = GMO technology, MEDS = medicines, SOL = solar energy, FARM = natural farming.

Furthermore, no main effect of valence (F(1,30) = .88, p = .356 η^2^_s_ = .028) or interaction between type and valence (F(5,150) = 1.27, p = .279 η^2^_s_ = .041) were observed. The effect of the electrode was observed (F(3, 90) = 47.53, p < .001η^2^_s_ = .613), however no interactions between electrode and other factors were found. The results can be found in Fig 5.

### Perceived expertise on presented issues

Participants were on average acquainted with most of the subjects (86%), with vaccines being the most recognized subject (100%) and nuclear energy the least recognized one (69%).

**Fig 6:**
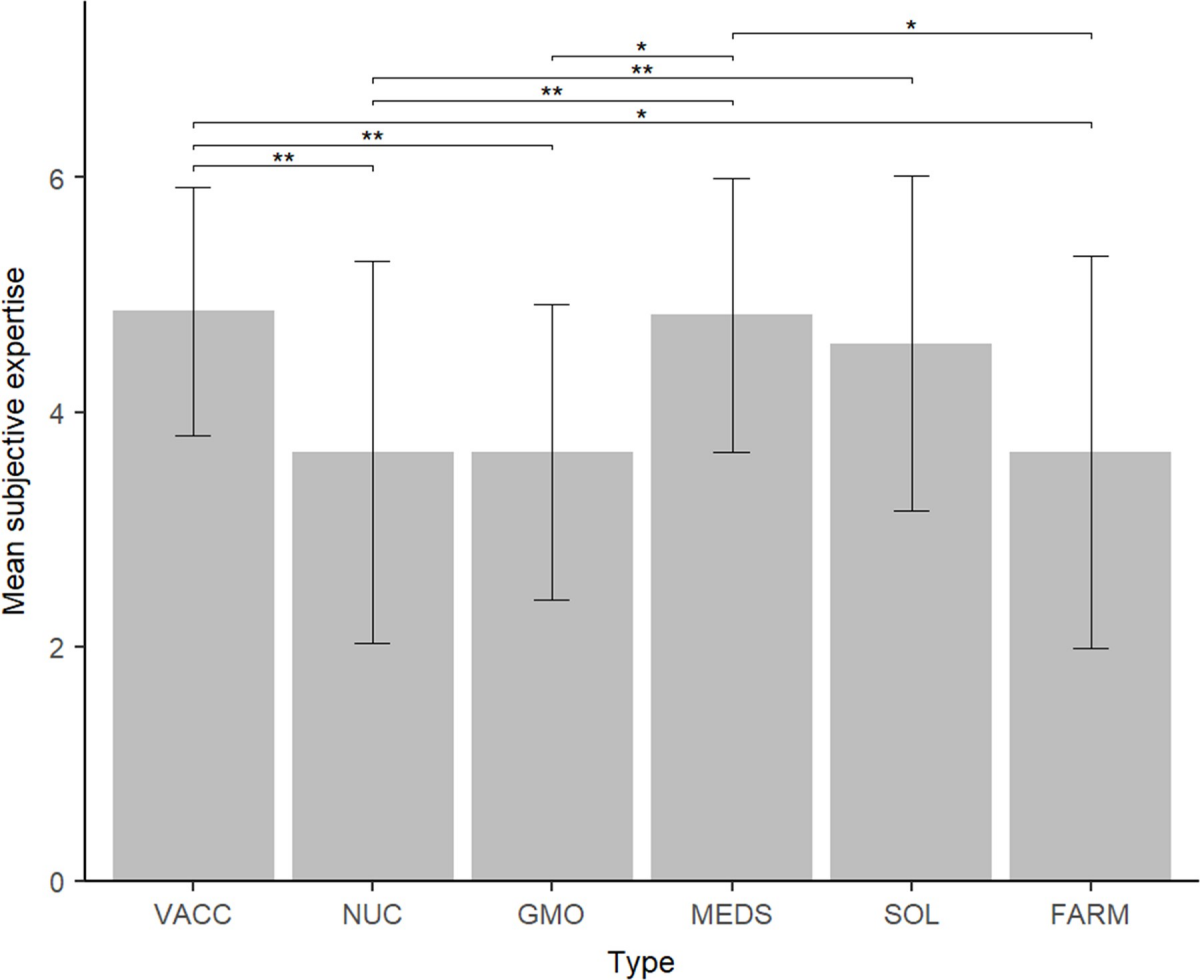
Rating of self-perceived expertise for six domains presented during the study. VACC = vaccination, NUC = nuclear energy, GMO = GMO technology, MEDS = medicines, SOL = solar energy, FARM = natural farming. Error bars represent standard deviation. *P* < .01, * *p* < .05.

Moreover, significant differences between declared expertise on different issues were found, F(5,140) = 7.57, p < .001 η^2^_s_ = .213). Bonferroni-corrected pairwise comparisons revealed that participants felt more knowledgeable about vaccines (4.86+/-1.06) and medicines (4.8+/-1.17) than about GMO (3.66+/-1.26), natural farming (3.66+/-1.67), and nuclear energy (3.66+/-1.63). They also felt more knowledgeable about solar energy (4.59+/-1.43) than about nuclear energy. The results can be found in Fig 6.

### Correlations between task-related variables

Self-perceived expertise was significantly correlated with task related responses for vaccines (positive words: rho = .458 p = .012), farming (negative words: rho = -.472 p = .010) and nuclear energy (positive words: rho = .678 p < .001; negative words: rho = .563 p = .001).

### Scientific Literacy Scale (SLS) and pseudoscience beliefs

Among four categories of pseudoscience claims listed in the online questionnaire, GMO-linked claims were seen as more likely to be truth (F(3, 69) = 6.45, p = .001 η^2^_s_ = .219; 2.93+/-.75) than claims linked to climate-change (2.37+/-.77; t = 3.88 p = .005), as well as trend-level more likely than psychological-claims (2.54+/-.60; t = 2.74 p = .070). However, investigation of this effect has shown that it was linked to mean response for GMO-related questions corresponding to the middle-point of the scale (‘Hard to say’), compared to climate-change statements, that were predominantly rated as untrue.

Pseudo-scientific beliefs have been strongly negatively correlated with task-responses for vaccines (positive words: rho = -.618 p = .001), medicines (positive words: rho = -.608 p = .002), GMO (positive words: rho = -.720 p < .001; negative words: rho = -.606 p = .002) and nuclear energy (positive words: rho = -552 p = .005; Bonferroni’s correction for 12 comparisons).

Finally, higher SLS accuracy was found to be positively correlated with task-responses for positive adjectives following nuclear energy (rho = .591 p = .002) cues (other correlations did not remain significant after Bonferroni’s correction for 12 comparisons).

## Discussion

The aim of the current study was to investigate attitudes toward selected science-related topics by using explicit (behavioral responses) and implicit (N400 amplitude) measures. Firstly, we have found an increased Late Positive Component (LPC) to words associated with topics which we deemed as linked to controversial topics compared to noncontroversial topics. LPC has been consistently found to be linked to the affective characteristics of the stimuli [42], thus this result may be indicative of the increased arousal elicited by controversial cues. Furthermore, as we observed in the analysis of the specific topics, this effect was particularly robust for GMO, which elicited increased LPC compared to all specific noncontroversial cues. This result is consistent with other studies [43].

The main finding of the current study is an increased amplitude of N400 in response to any target words following the prime linked to only one of the topics (GMO) included in the study. Similar effects were not found for other scientific issues included among prime words. Furthermore, no significant effects were observed at the level of the pooled analysis (controversial vs noncontroversial topics), which highlights the importance of taking into consideration specific topics while investigating attitudes toward science. Furthermore, no effect of valence of the presented words was found for GMO, i.e. N400 enhancement was observed for both positive and negative words, which suggest that participants perceived this issue in more ambiguous terms, rather than had any specific negative or positive bias toward this particular issue. This notion is substantiated by behavioral responses. Participants perceived positively-valenced target words as predominantly congruent and negatively-valenced target words as incongruent with primes (effects ranging from 65.9% of positive reactions for nuclear energy to 80.4% for vaccines). This effect was significantly decreased for GMO, where only 57.1% of positive reactions were found. Finally, a similar pattern of more ambiguous responding was found in a subgroup of participants who completed a post-hoc questionnaire assessment–participants have rated GMO-related pseudo-scientific statements with less degree of certainty than climate change statements.

Previous studies that utilized N400 to investigate stereotypes have interpreted larger amplitudes of this potential in response to stereotype-incongruent pairs of words as evidence for increased conflict monitoring and resolution processes [30, 31]. However, as conflict resolution and mismatch detection are not among the functions typically associated with functional significance of N400, alternative interpretations of these effects can be discussed. Furthermore, those studies did not include the measure of semantic N400 effects for strictly semantic processes, which could obscure the actual magnitude of the obtained results. Here, we have found no significant N400 effects for most of the categories included in the current study, which is in line with behavioral responses showing predominantly positive attitudes towards most of the science related issues. Taken together, the findings of the current study suggest that N400 deflection in this case may suggest a lack of a definitive stand and ambiguous perception of this specific category, rather than an antiscience stand in our participants. Mandatory labelling of GMO products in Western countries and advertising strategies based on the ‘GMO-free’ assumption, have been linked to the limited understanding and misconceptions about GMO practices [44]. The steady rise in positive attitudes towards GMO in the EU [45] combined with the constant disinformation shared in social media [46] can create a sense of confusion around the subject. A study with Polish school adolescents has found a relatively low level of knowledge about GMO [47]. Another study on a Polish sample found the majority of participants to admit that their understanding of GMO technology was insufficient [48]. Thus, those findings support the notion that this particular category may be less understood than other categories included in the current study. This interpretation, suggesting that N400 may be modulated by the lack of information about the certain scientific topics, is also strongly supported by previous studies which have shown that this potential may be used for tracking comprehender’s world knowledge [for a review of the studies, see: 49].

Interestingly, a similar pattern of behavioral responses was observed with regard to nuclear energy during the task: participants rated nuclear energy less favorably than vaccines, and they assessed their own expertise on the issue of nuclear energy as inferior compared to vaccines, medicines and solar energy. At the same time, the amplitude of the N400 waveform to nuclear energy was intermediate and did not significantly differ neither from the amplitudes observed for primes producing the smallest effect (vaccines, solar energy), nor from the amplitude observed for the prime producing the largest effect (GMO). Additionally, during the post-hoc assessment, participants rated pseudoscience claims associated with climate change as less likely to be true compared to GMO-related claims. However, the claims presented in the post-hoc questionnaire did not directly refer to nuclear energy, which may explain the dissociation between within task behavior and post-hoc assessment. A recent meta-analysis of 34 empirical studies with overall 32 938 participants has shown that acceptance of nuclear energy is shaped by a number of factors, related both to participants’ characteristics (sex, education, knowledge, trust) and public perception of benefits, risks and costs of nuclear energy [50]. This way, further stratification of the groups of participants may be necessary to reliably establish the pattern of responses to nuclear energy.

Finally, despite our initial predictions, our participants have shown very positive attitudes toward vaccines, which were perceived in a more positive light than medicines. While the emergence of the anti-vaccine movement may be one of the most important phenomena underlying the current antiscience stand [51], it has also led to the widespread debate over the safety of the vaccines. Thus, paradoxically, the increased public scrutiny over this issue may have increased the positive beliefs about vaccines safety and efficiency among some part of the population, as a result of the exposure to scientific knowledge about vaccines [52, 53]. However, it is worth noting that the study was completed before the COVID-19 pandemics, which significantly distorted the reception of vaccines in many ways, as well as disrupted the standard vaccines’ rollout [54]. It increased the awareness of how vaccines work in the general population, but also fueled various anti-vaxxer sentiments and anti-scientific movements, and the overall result on vaccine hesitancy remains unknown [55].

The pandemic has shown that the acceptance of COVID-19 vaccine is associated with health literacy and the ability to detect fake news [56]. The results of our study also highlight the importance of knowledge for attitudes towards ambiguous scientific topics. Similarly to previous work [21], the participants’ scientific literacy was positively related to perceived congruence of positive words with the least known topic (i.e. nuclear energy), while the belief in pseudo-scientific claims was negatively linked to positive task-responses to vaccines, medicines, GMO and nuclear energy. The association between N400 amplitude for positive GMO-cues and perceived understanding of the subject can constitute additional evidence that implicit ambiguity of scientific issues decreases with higher subjective knowledge.

The COVID-19 pandemic has significantly affected the anti-scientific movement. On the one hand, it may have increased the general awareness of human viruses, transmissivity, or herd immunity issues, and convinced some of the uninvolved population to the need for vaccinations. On the other hand, the COVID-19 pandemic in itself has become a cradle of various conspiracy theories, disinformation wars, and the rise of anti-vaxxer groups [57, 58]. Our study, conducted just before the first lockdowns, offers a unique insight into the stereotyping of various scientifically grounded notions and phenomena, such as climate change, vaccines, or GMO. The results of the study suggest that N400 may be used for tracking implicit processes associated with uncertainty towards specific scientific issues. Furthermore, by complimenting overt participants’ responses, it may provide better understanding factors shaping one’s attitude toward science (e.g. domain-specific expertise or general scientific literacy).

While the current study shows the potential benefits of using the ERP for investigation of attitudes toward scientific issues, it is limited by its design. Firstly, we have included undergraduate students who showed generally positive attitude toward all presented issues, thus the current results should be extended by inclusion of a more general population of subjects, or, particularly, individuals presenting an overly antiscience stand, as it was not feasible with the current convenience sample to account for between-subject variability in the behavioral and neural response to the scientific issues. Furthermore, we have utilized an explicit paradigm to evoke N400 changes, thus further studies can use more sophisticated paradigms that may provide more subtle cues, e.g. Implicit Association Test [59]. Furthermore, while multiple lines of the evidence suggest that the increased N400 amplitude to GMO may be linked to the increased ambiguity towards this issue, we did not control the frequency of use of prime words in Polish, thus it cannot be clarified whether this effect cannot be still linked to the psycholinguistic characteristics of the topic words. Finally, while we assumed that some of the issues included in the study may be more polarizing than similar issues that do not elicit public debate, the pattern of the responses observed in our participants was not congruent with this approach (e.g. vaccines were perceived more positively than medicines), thus we abstained from pooling primes into larger factors (e.g. controversial vs noncontroversial). Unfortunately, this action resulted in including a small (n = 20) amount of trials in each category, thus further studies in the area should a priori assume that specific categories need to be directly compared and warrant better powered paradigms for such aims. Additionally, we did not precede the EEG study with behavioral study that would allow examining the level of perceived congruence between specific adjectives and topics included in the study, we based it on the assumption that, given the overall positive attitudes to all of the presented topics, the positive adjectives should be perceived as congruent while the negative adjectives should be seen as incongruent with topics included in the current study. However, further studies should provide a more tailored selection of the adjectives for each topic, as the congruence is the main factor driving the N400 effects.

## Supporting information

S1 Table List of word pairs from the classic N400 paradigm (translated from Polish). (DOCX)
