# Supplementary material for: Climate change, vaccines, GMO: The N400 effect as a marker of attitudes toward scientific issues
Source: PLoS One. 2022 Oct 6;17(10):e0273346. doi: 10.1371/journal.pone.0273346 (PMC9536546; doi:10.1371/journal.pone.0273346)
Supplement: S1 Table — (DOCX) [file pone.0273346.s001.docx]

List of word pairs from the classic N400 paradigm (translated from Polish)

| **Cue** | **Congruent** | **Incongruent** |
| --- | --- | --- |
| cottage | country | corrosive |
| bread | stale | soaring |
| disease | infectious | empty |
| hole | deep | sour |
| house | family | fat |
| carpet | soft | tart |
| child | small | miry |
| mountain | high | thin |
| food | tasty | rapid |
| bath | hot | prickly |
| cabbage | fermented | open |
| ice | cold | loud |
| stem | green | swift |
| honey | sweet | short |
| hammer | iron | brisk |
| butterfly | colorful | solid |
| sky | blue | fast |
| scissors | sharp | boiling |
| ocean | big | tiny |
| window | open | spry |
| sheep | black | strict |
| stove | warm | faint |
| work | hard | yellow |
| order | military | wet |
| snow | white | lively |
| salt | salty | noisy |
| light | bright | flat |
| street | long | brittle |
| Earth | round | slender |
| turtle | slow | dense |
